# Management of Glomus Tumors: Experience From a Tertiary Care Centre in India

**DOI:** 10.7759/cureus.86227

**Published:** 2025-06-17

**Authors:** Nitin Choudhary, Archi Gupta, Akash Narangyal, Bias Dev, Sanjeev Gupta

**Affiliations:** 1 Orthopedics, Government Hospital for Bone and Joint Surgery, Jammu, IND; 2 Radiology, Government Medical College and Hospital, Jammu, Jammu, IND; 3 Orthopedics, Government Medical College and Hospital, Jammu, Jammu, IND

**Keywords:** delayed diagnosis, glomus tumour, hand tumour, histological features, soft-tissue tumour

## Abstract

Background: Glomus tumors are uncommon soft tissue tumors that typically develop in the distal extremities, especially the finger's subungual area. Pain, soreness, and temperature intolerance, particularly cold sensitivity, make up its traditional clinical triad. There is a relative rarity in the literature on this topic, and we have analyzed and thereby discussed our experience in these cases.

Methods: A retrospective cross-sectional study was performed at a tertiary care centre in Jammu, India. All patients diagnosed with glomus tumor from October 2022 to August 2024 were included in the study. Conventional radiographs, ultrasonography (USG), and MRI were the investigation modalities used for diagnosis. Tumors excised were sent for histopathological examination (HPE) for pathological diagnosis.

Results: A total of 11 cases of glomus tumor were diagnosed from October 2022 to August 2024, and the relevant demographic and clinical data were reported. Out of 11 cases, nine (81.8%) were located in the nail bed, while two (18.2%) were located in the volar pulp. Pain was present in almost all the cases, while a lump was visible in only one case of volar pulp tumor. There was a considerable delay in diagnosis in the majority of cases. There were no cases of recurrence.

Conclusion: Due to their rarity, glomus tumors are frequently overlooked by clinicians due to a lack of a strong index of suspicion, which typically results in treatment being delayed. Once identified, glomus tumors can be effectively treated with complete excision.

## Introduction

Glomus tumors are relatively rare, accounting for about 1-5% of all hand tumors. Even while they can form anywhere on the body, they usually do so in the upper extremities, specifically in the subungual regions. Tenderness, cold intolerance, and acute paroxysmal pain in the fingertips are their defining characteristics [1,2]. Glomus tumors originate from the glomus body, which is a component of the dermal thermoregulation system. They were first described by Wood back in 1812 [3]. In 1924, Masson referred to glomus tumors as tumors that occur in the neuro-myo-arterial body [4]. Glomus cells, part of the body’s thermoregulation system, are responsible for managing blood flow through small arteriovenous shunts known as glomus bodies. When these cells proliferate abnormally, they form glomus tumors. These growths are usually small and well-circumscribed but can lead to severe pain and sensitivity, especially in colder environments [5]. Despite being non-cancerous, glomus tumors often present a challenging diagnostic and therapeutic scenario due to their unique characteristics.

## Materials and methods

This retrospective cross-sectional study included patients diagnosed with glomus tumors of the hand at the Government Medical College and Hospital, Jammu, India, from October 2022 to August 2024. All patients presented with chief complaints of hand/digit pain, which was not relieved on taking analgesics and was tender to touch, with exacerbation in a cold environment. All patients were seen in peripheral clinics for the same, however, the case was not diagnosed and hence reported to the tertiary centre. A total of 14 patients were initially included in the study; however, two patients were lost in follow-up, and one patient’s documents/records were not recovered; hence, our final study included a total of 11 patients.

**Figure 1:**
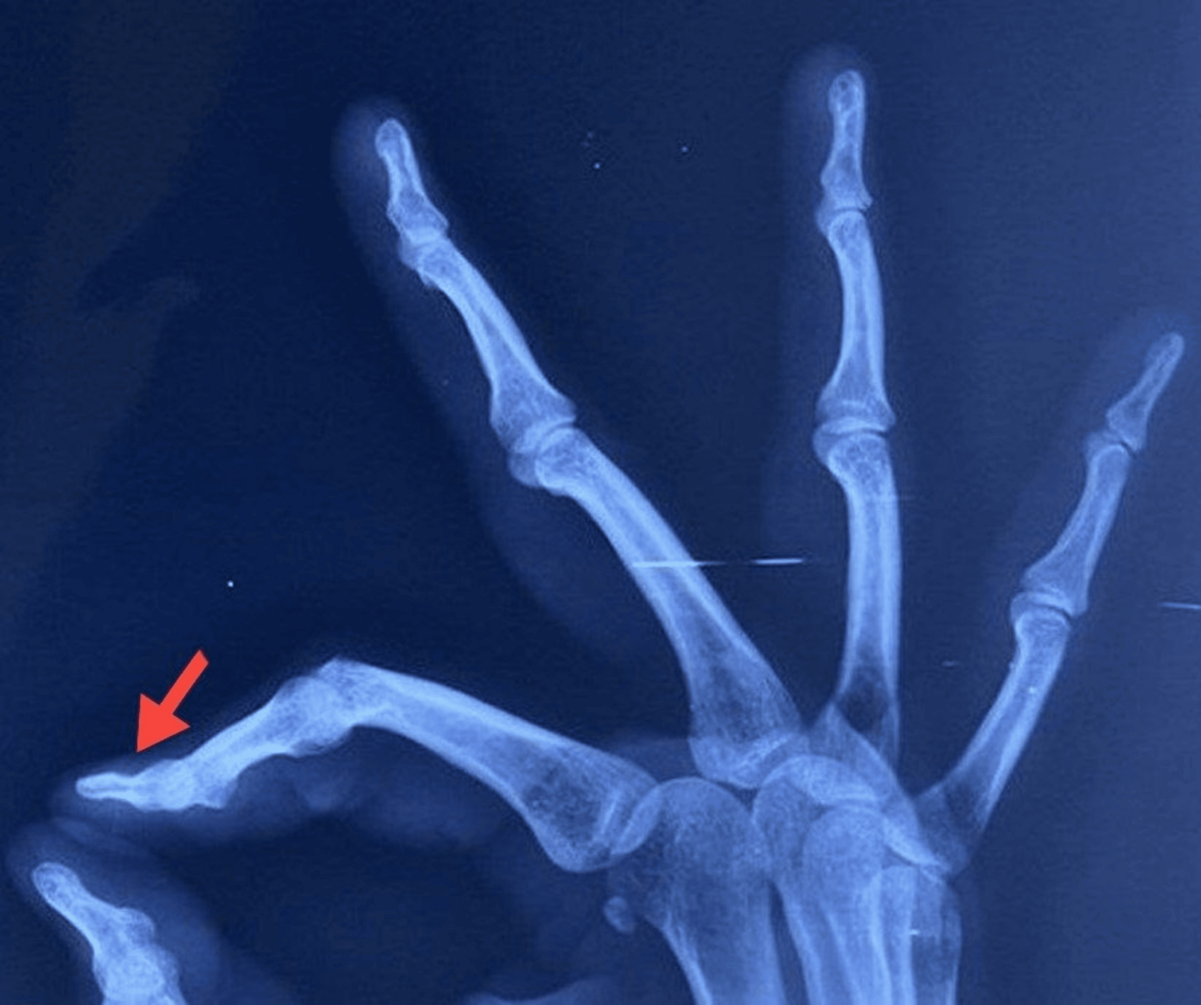
Lateral radiograph showing a scalloping effect on the bone caused by a glomus tumor (red arrow).

**Figure 2:**
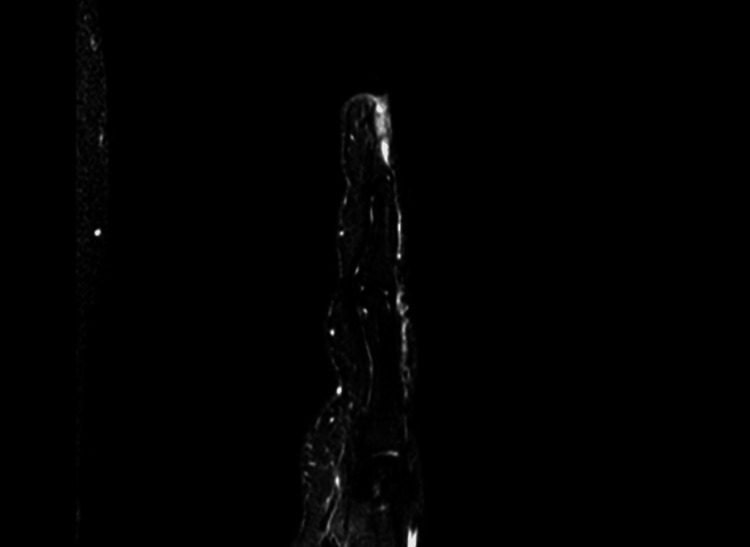
Sagittal MRI image showing the presence of a glomus tumor.

**Figure 3:**
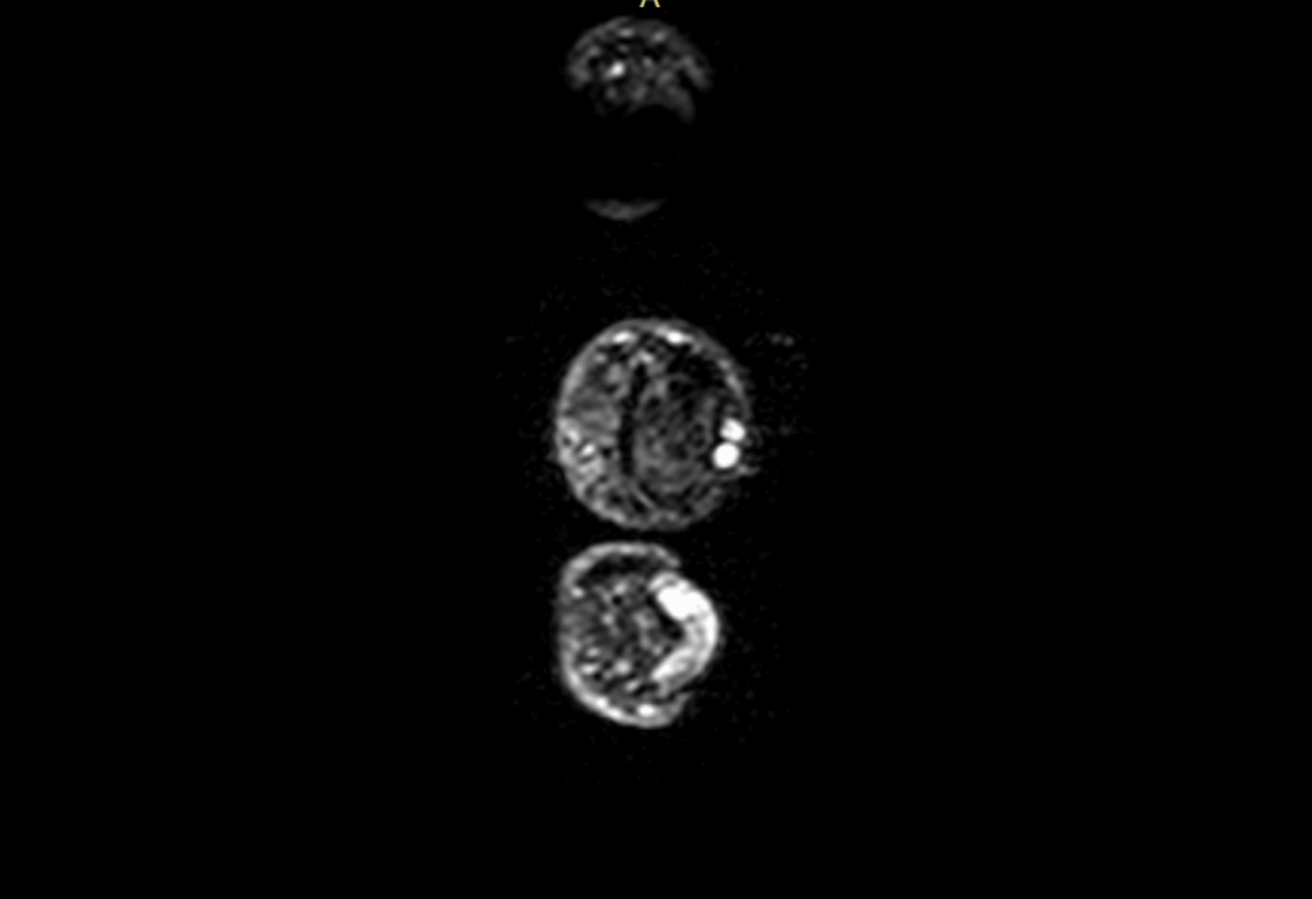
Axial MRI image showing a glomus tumor.

All patients were initially sent for plain X-ray radiographs (Figure 1) followed by ultrasonography. Patients detected with glomus tumor were sent for magnetic resonance imaging (MRI) (Figures 2, 3) for final definitive diagnosis. The patients were operated under ring block of the involved digit and were discharged the same day. All excised tumors were sent for histopathological examination (HPE) confirmation.

**Figure 4:**
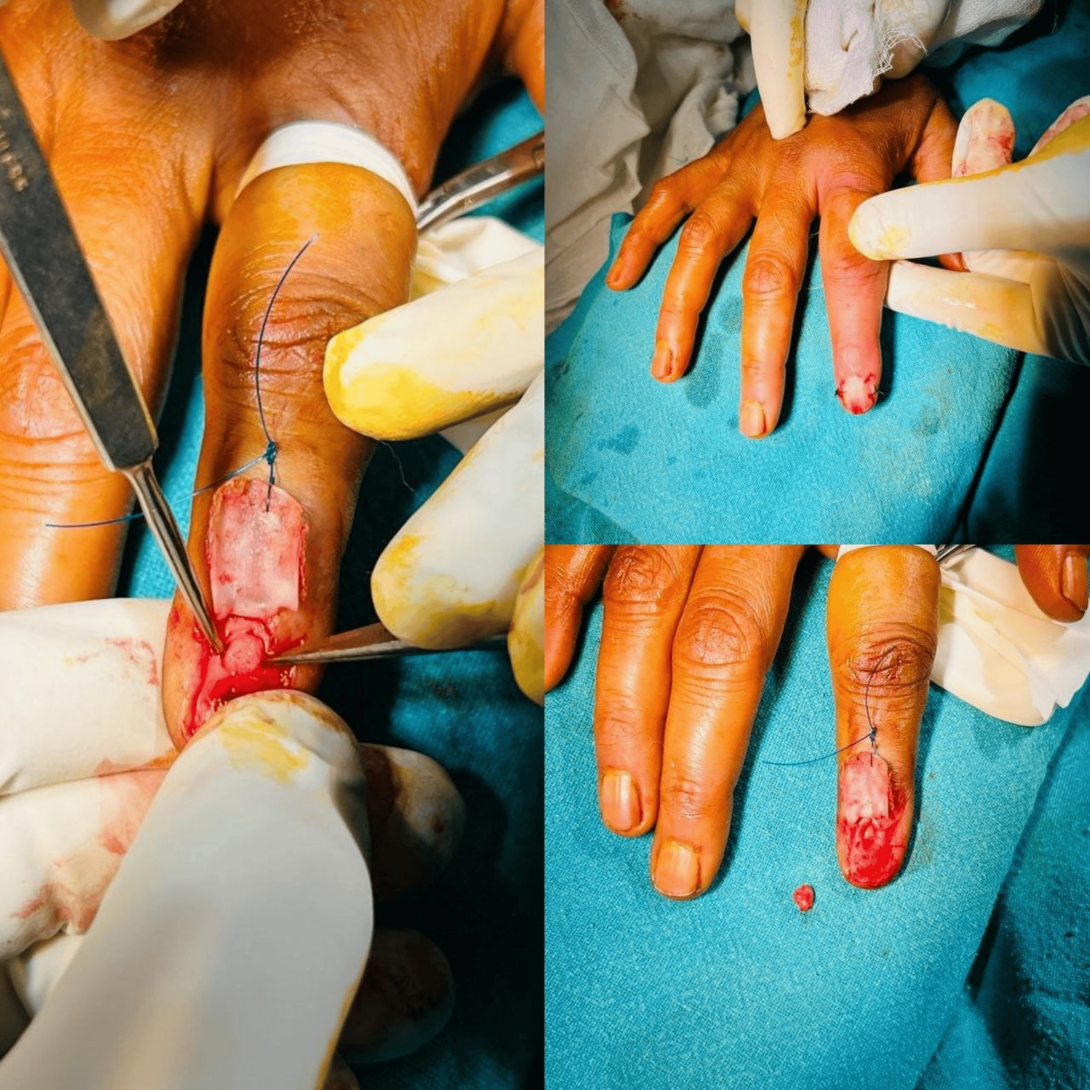
En-masse removal of the tumor using the trans-ungual approach under digital block anesthesia, followed by closure.

For the excision of hand glomus tumors, three surgical approaches are commonly described: trans-ungual, lateral subperiosteal, and volar. We used a trans-ungual approach for tumours of the nail bed, while the volar approach was used for volar tumours. The surgery was performed under ring block (digital anaesthesia), and a finger glove tourniquet was used (Figure 4).

## Results

A total of 11 cases were included in the study. Of 11 patients, nine were females (81.8%) and two were males (18.2%). The mean age of presentation for both genders was 35 years. There was a history of pain for a long duration (mean: 21.1 months), with consultations for the same prior to reporting to the tertiary centre. All surgeries were done as outpatient procedures, and patients were discharged the same day with coverage of oral antibiotics and good analgesia. The samples sent for histopathological diagnosis affirmed our diagnosis. In a low-power field, the tumors were coated in fibrous capsules that were comparatively well-defined. Regularly shaped epitheloid cells were arranged in one or more layers around the dilated, thin-walled blood arteries. Epitheloid cells with a spherical or oval nucleus and essentially no mitotic signs were seen in a high-power field. Pain was relieved the very next day of surgery. There was no wound-related complication in any of the patients. Patients were followed up for a minimum of nine months. There was no case of recurrence of pain or any of the other initial presenting symptoms in any of the patients (Table 1).

**Table 1:** Patient demographics and tumor characteristics. M: male; F: female

Patient	Sex	Age (in years)	Location	Time to presentation (in months)	Follow-up period (in months)	Complication	Recurrence
1	F	27	Nail bed	18	13	Nil	No
2	F	37	Nail bed	20	11	Nil	No
3	F	41	Nail bed	28	14	Nil	No
4	F	36	Volar pulp	27	10	Nil	No
5	F	38	Nail bed	12	9	Nil	No
6	F	34	Nail bed	27	14	Nil	No
7	M	29	Nail bed	28	18	Nil	No
8	F	42	Nail bed	15	12	Nil	No
9	F	39	Volar pulp	24	11	Nil	No
10	M	34	Nail bed	15	9	Nil	No
11	F	28	Nail bed	18	16	Nil	No

## Discussion

Situated in the reticular layer of the dermis, a glomus body is a unique arterio-venous anastomosis accountable for thermoregulation. The normal glomus body, consisting of glomus cells that are distorted smooth muscle cells found in the walls of the Sucquet-Hoyer canal, is the source of glomus tumors. They typically start in the skin, but they have also been known to appear in internal organs like the stomach, lung, trachea, and bone, as well as mucous membranes [6,7]. In the hand subungual region (most common), the lateral side of the digits and the palms are the most frequently affected sites.

Because glomus tumors typically arise in the extremities, particularly the digits of the upper extremities, hand or orthopaedic surgeons encounter them frequently [8]. They are also discovered in other places like the wrist, toe, hand, and knee, which is consistent with a group of 56 extra-digital glomus tumors that Schiefer et al. reported, of which 91% were detected in the extremities [9]. Glomus tumors are more common in women between the ages of 20-40, which is consistent with the findings of our study. However, males have a greater predisposition for glomus tumors at extra-digital sites [7].

Almost all patients in our study had extreme localized pain, which is consistent with their clinical presentation in the literature. There was a considerable delay in diagnosis from the time of the index complaint in most case series in the literature [7,8]. Therefore, a high index of suspicion is required in diagnosing these tumors.

Several studies have discussed radiological investigations as a supplement to clinical suspicion in forming a final diagnosis. Despite being a soft tissue tumor, glomus tumors can cause nearby bone to scallop, which can be seen on plain radiographs [10,11]. Ultrasound has been used to diagnose glomus tumor, owing to its hyper-vascular nature [10,12], and was considered the standard radiological modality in these cases. The glomus tumor, which typically manifests as a well-circumscribed lump that is hypo-intense on T1-weighted imaging and hyper-intense on T2-weighted images, can be diagnosed with an MRI scan, which is recently being commonly used for definitive diagnosis [8,11]. We, in our study, used both these modalities for diagnosing our patients.

Complete removal is thought to be curative and frequently has positive results [13]. All cases in our series had a benign glomus tumor identified by histology. While some studies claim a recurrence incidence of up to 30%, the majority of research in the literature also reports a low rate of recurrence, with extra-digital glomus tumors accounting for 85% of cases having recurrence [11,13]. A case series conducted by Sacchetti et al. in 2019 discussed a case of neoplastic distal forearm glomus tumor managed by excision along with adjuvant therapy [14]. The authors agreed that more research with longer follow-up periods will provide a more complete picture of the recurrence rate and clinical outcomes, given that the average follow-up period in this study was just 5.8 months. However, the authors across most studies in the literature have a general consensus that hand glomus tumors are benign tumors, which are treated best by complete excision with minimal/no recurrence rate [15-17].

The limiting factors in our study were a smaller number of cases, as a large number of cases or multi-institutional trials would have given a better epidemiological picture. While the minimum follow-up period in our study was nine months and the maximum follow-up was 18 months, having longer follow-up periods would have given a better and clearer picture on account of disease recurrence.

## Conclusions

Hand glomus tumors, while benign, can significantly affect patients’ quality of life due to their painful and disruptive symptoms. It may be readily missed by clinicians because of its rarity and its being frequently clinically insignificant except for the presence of severe pain, which prolongs the time it takes to arrive at the correct diagnosis. Reducing discomfort and averting recurrence need early detection and effective treatment, with surgical excision offering negligible complications and minimal recurrences in expert hands.
